# Automated Fall Detection Algorithm With Global Trigger Tool, Incident Reports, Manual Chart Review, and Patient-Reported Falls: Algorithm Development and Validation With a Retrospective Diagnostic Accuracy Study

**DOI:** 10.2196/19516

**Published:** 2020-09-21

**Authors:** Elisa Dolci, Barbara Schärer, Nicole Grossmann, Sarah Naima Musy, Franziska Zúñiga, Stefanie Bachnick, Michael Simon

**Affiliations:** 1 MediZentrum Täuffelen Täuffelen Switzerland; 2 Nursing & Midwifery Research Unit Inselspital Bern University Hospital Bern Switzerland; 3 Department of General Internal Medicine Inselspital Bern University Hospital Bern Switzerland; 4 Institute of Nursing Science, Department of Public Health Faculty of Medicine University of Basel Basel Switzerland

**Keywords:** falls, adverse event, harm, algorithm, natural language processing

## Abstract

**Background:**

Falls are common adverse events in hospitals, frequently leading to additional health costs due to prolonged stays and extra care. Therefore, reliable fall detection is vital to develop and test fall prevention strategies. However, conventional methods—voluntary incident reports and manual chart reviews—are error-prone and time consuming, respectively. Using a search algorithm to examine patients’ electronic health record data and flag fall indicators offers an inexpensive, sensitive, cost-effective alternative.

**Objective:**

This study’s purpose was to develop a fall detection algorithm for use with electronic health record data, then to evaluate it alongside the Global Trigger Tool, incident reports, a manual chart review, and patient-reported falls.

**Methods:**

Conducted on 2 campuses of a large hospital system in Switzerland, this retrospective diagnostic accuracy study consisted of 2 substudies: the first, targeting 240 patients, for algorithm development and the second, targeting 298 patients, for validation. In the development study, we compared the new algorithm’s in-hospital fall rates with those indicated by the Global Trigger Tool and incident reports; in the validation study, we compared the algorithm’s in-hospital fall rates with those from patient-reported falls and manual chart review. We compared the various methods by calculating sensitivity, specificity, and predictive values.

**Results:**

Twenty in-hospital falls were discovered in the development study sample. Of these, the algorithm detected 19 (sensitivity 95%), the Global Trigger Tool detected 18 (90%), and incident reports detected 14 (67%). Of the 15 falls found in the validation sample, the algorithm identified all 15 (100%), the manual chart review identified 14 (93%), and the patient-reported fall measure identified 5 (33%). Owing to relatively high numbers of false positives based on falls present on admission, the algorithm’s positive predictive values were 50% (development sample) and 47% (validation sample). Instead of requiring 10 minutes per case for a full manual review or 20 minutes to apply the Global Trigger Tool, the algorithm requires only a few seconds, after which only the positive results (roughly 11% of the full case number) require review.

**Conclusions:**

The newly developed electronic health record algorithm demonstrated very high sensitivity for fall detection. Applied in near real time, the algorithm can record in-hospital falls events effectively and help to develop and test fall prevention measures.

## Introduction

Falls are among the most common adverse events in hospitals [1]. For example, US hospitals report fall rates per 1000 patient days ranging from 3.3 to 11.5 [1], while Swiss studies have reported rates between 2.2 and 8.9 [2,3]. A fall is defined as “an unexpected event in which the person comes to rest on the ground, floor or other lower level [4].” Approximately 25% of in-hospital falls lead to injuries, the most serious of which are fractures and intracranial hemorrhages [1]. Increasing disability-related dependence, length of stay, and care costs make falls a major burden, not only for the affected patients, but for the entire health care system [5].

Therefore, the development, evaluation, and improvement of interventions to prevent falls are a high-priority for health researchers. However, quick, accurate, and cost-effective fall detection methods are needed to provide reliable and robust fall data; currently, no such method is available.

The 3 most common fall detection methods are voluntary incident reporting, chart reviews, and patient self-reports [6]. Voluntary incident reports are provided by frontline staff directly, often nurses, involved in falls or in the action leading up to it [6-8]. Traditional chart reviews consist of reading the full patient records. The Global Trigger Tool is a retrospective chart review method developed by the Institute for Healthcare Improvement. It is widely used internationally for detecting adverse events and uses so-called triggers (ie, key elements that help reviewers to identify potential adverse events including falls) [9-12]. Finally, in Switzerland, prevalence data of patient-reported in-hospital falls are recorded based on the LPZ method (*Landelijke Prevalentiemeting Zorgproblemen*, National Prevalence Measurement of Quality of Care) [13]. Unlike incident reports, where staff fill forms when a fall occurs, this measure is based on a self-reported questionnaire or retrospective interview by hospital staff (30-day period). Since 2009, this measurement has been conducted annually on a single day by the ANQ (Swiss National Association for Quality Development in Hospitals and Clinics) in almost all Swiss acute care hospitals [14].

Each of these methods is limited in important ways. Nurse voluntary incident reports are prone to underreporting or nonreporting [8]. Chart review is time consuming and costly. And the LPZ/ANQ patient reports are affected both by underreporting and by the lack of flexibility regarding their timing. These limitations make a quick, accurate, and timely fall detection system highly desirable.

One very promising target for research is hospitals’ electronic health records. As digital databases, these offer the opportunity to develop automated detection algorithms. In addition to being inexpensive to use, such methods would potentially be both highly sensitive and fast enough to deliver real-time or near real-time data on adverse events [6,12,15]. Setting the technical advantages of electronic health record–based adverse event detection aside, research in this area is still relatively new and not systematically under study. In their review, Musy et al [16] found a broad interstudy variation in reported adverse event prevalence and positive predictive value, which led to difficulties regarding interpretation. To improve quality, they see the need for adequate reporting of future adverse event detection studies [16].

Because of these and other potential advantages, algorithms for adverse event detection are being developed more and more [17-20]. To our knowledge, only one—in Japan—has used electronic health record data for fall detection—with mixed results [21]. The algorithm, which used natural language processing to read medical professionals’ chart notes for a sample of 1204 patients, was highly sensitive regarding fall detection (100%); however, its positive predictive value was very low (6%) [21]. Therefore, this study’s goal was to develop and validate an electronic health record–based fall detection algorithm (using the given German-language electronic health record systems), then to test its diagnostic accuracy against manual chart review and patients’ reports of falls.

## Methods

### Design (Study 1 and Study 2)

This retrospective diagnostic accuracy study consisted of 2 parts: the first, for algorithm development and the second, for validation. For the development of the electronic health record fall detection algorithm, we used falls identified through the Global Trigger Tool in a previous study [22] along with incident reports for comparison. To validate the algorithm, we collected additional data to compare the algorithm against falls identified through the manual chart review of electronic health records (“Global Trigger Tool for falls only”) and patient-reported falls based on the LPZ/ANQ measure.

### Setting (Study 1 and Study 2)

This study was conducted in one large Swiss university hospital and in one rural hospital belonging to the same hospital system in the German part of Switzerland. From the university hospital, 2 departments participated: Internal Medicine (110 beds, approximately 4600 admissions per year, average length of stay 6.5 days) and Orthopedics and Plastic Surgery (59 beds, approximately 2400 admissions per year, average length of stay 7.4 days). The rural hospital’s general medicine, general surgery, visceral surgery, traumatology, and orthopedics units participated (totaling 72 beds, approximately 5200 admissions per year, average length of stay 5.4 days). Because internal medicine and orthopedics departments treat older people with chronic diseases, which are risk factors for falls, these departments have relatively high fall rates. The university hospital introduced electronic health records in 2011, while the rural hospital introduced electronic health records in 2010. Until September 2017, the 2 facilities had separate electronic health record systems but with similar internal databases. The algorithm development study occurred only in the university hospital’s Internal Medicine department; the validation was performed in the 2 university hospital departments and in all the participating departments of the rural hospital.

### Study 1: Algorithm Development

#### Sample and Sampling

The algorithm was developed by one of the first authors (BS), using data from a previous Global Trigger Tool study [22]. That study’s [22] sample consisted of patients admitted to the Internal Medicine department between September 1, 2016 and August 31, 2017. Further inclusion criteria were (1) adult patients (aged ≥18 years), (2) closed and completed patient record, and (3) inpatients with a length of stay of at least 24 hours. From the eligible patients’ data sets, we randomly selected 240. The first 120 (the development data set) were used to develop the algorithm; the remaining 120 (the testing data set) were used to validate the algorithm. Because this was a diagnostic accuracy study, no formal power analysis for sample size was conducted. However, for the Global Trigger Tool study [22] and an expected overall adverse event rate of 12.3% as detected by Soop et al [23], a sample size of 240 gives a 95% confidence interval of 8.9%-16.7%. Electronic patient records (n=30) from hospitalized patients were randomly selected each month and checked for eligibility, including their general consent, by one reviewer. Of the 30 records, the first 20 that were eligible were used for chart review each month.

#### Data Collection and Management

Three health care professionals completed the Global Trigger Tool review: 2 nurses as primary reviewers (with 5 years of clinical experience and knowledge of the electronic health record) and a physician (with 10 years of clinical experience). As preparation, reviewers read the Health Care Improvement handbook for the Global Trigger Tool and underwent training provided on the website [9]. Furthermore, the primary reviewers practiced on 15 patient charts, 5 of which were discussed with the physician. The interrater reliability (Cohen κ) on the number of adverse events between the primary reviewers was 0.96 and between the primary reviewers and the physician was 0.98.

#### Variables and Measurement

In order to describe the sample, we also extracted basic patient characteristics, such as age, gender, length of stay, and primary diagnosis, from the electronic health records. All 4 variables were also considered risk factors for falls [3]. We focused on in-hospital fall rates recorded by our algorithm, the Global Trigger Tool, and voluntary incident reports (Table 1). For the in-hospital falls variable we used Hauer et al’s definition [4], which includes 3 categories: assisted falls (eg, when the patient begins to fall and is assisted to the ground by another person); unassisted falls; and falls resulting from syncopes, epileptic seizures, strokes, and hypoglycemia. All types of accidents (eg, sporting, road traffic, work-related) leading to falls as the cause for hospitalization were excluded. Electronic health record reviews using the Global Trigger Tool were limited to 20 minutes. Multimedia Appendix 1 presents details of these variables.

**Table 1:** Variables of the algorithm development and validation study.

Variable	Description	Development: Method as data source	Validation: Method as data source
Age	Years at the time of admission	Global Trigger Tool	LPZ/ANQ^a^ measure
Gender	Female or male sex	Global Trigger Tool	LPZ/ANQ measure
Length of stay	Number of days in the hospital	Global Trigger Tool	Manual chart review
Primary diagnose	Cardiac, musculoskeletal, endocrinologic, gastrointestinal, pulmonary, infectious, neurological, psychiatric, cancer, dementia	Global Trigger Tool	LPZ/ANQ measure
Presence of fall	Yes or no	Algorithm, Global Trigger Tool, voluntary incident reports	Algorithm, manual chart review, LPZ/ANQ-measure
Fall rates	Number of falls	Algorithm, Global Trigger Tool, voluntary incident reports	Algorithm, manual chart review, LPZ/ANQ measure
Time for data collection	Time for data collection in hours	Global Trigger Tool	Manual chart review

^a^LPZ/ANQ: Landelijke Prevalentiemeting Zorgproblemen/Swiss National Association For Quality Development in Hospitals and Clinics.

#### Algorithm Development

**Figure 1:**
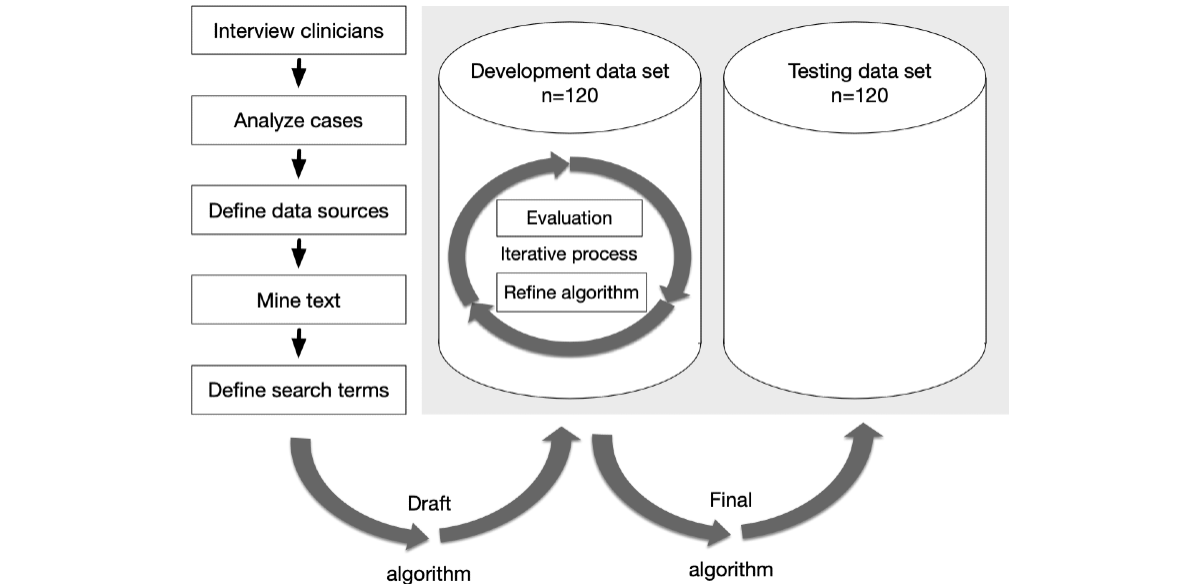
Algorithm development process.

For the algorithm development, a positive test case was comprehensively analyzed to identify appropriate data sources within the electronic health record system (Figure 1). Nurses’ and physicians’ narrative progress notes proved the most promising data source. It was important to take both sets of progress notes into consideration, as fall events were not always mentioned by both physicians and nurses. We compiled a list of fall-related terms—*fall*, *fell*, *slip*, *floor*, etc, which would be used to describe an event. Common terms used in the record were *am Boden* (*on the floor*), *ausgerutscht* (*slipped*), *Sturz/Stürze* (*fall/falls*), *Synkope/synkopiert* (*collapse/collapsed*). After identifying the most fall-specific terms, we transformed the words into search strings to build the algorithm. Extraction was performed using the widely used structured query language (SQL) for Oracle Databases.

To distinguish true positives from false positives, algorithm results were compared to those of the manual Global Trigger Tool study in the development set. For false positives, a comprehensive investigation was performed to identify misleading terms. For instance, the German term *Boden* (*floor*) was used to report that a patient has been found on the floor. However, the term was also used in other contexts, notably *Bodenbett* (low-level bed used to reduce the risk of bed fall injuries). As this resulted in a large number of false positive cases, *Bodenbett* was added to the exclusion criterion in the query.

If an event identified by the manual Global Trigger Tool study was not found by the algorithm, progress notes were analyzed comprehensively to identify further search terms. Since first iterations revealed difficulties distinguishing inpatient falls from fall injuries present on admission, we used a text-mining approach. This identified terms related to emergency situations, for example, *emergency* or *ambulance*, or the term *at home* as indicators of preadmission events. Fall events could also result from critical events (eg, loss of consciousness) or accidents, either of which can lead to emergency hospital admissions on their own.

Through the process described above, selection criteria were defined for patient records (Multimedia Appendix 2). These were used in the algorithm. One of our first steps was to query records indicating the presence of fall events; in subsequent steps we excluded events present on admission. The algorithm’s accuracy was compared to the manual Global Trigger Tool results, then optimized by iteratively testing it with the development set of electronic health record medical charts (Figure 1).

### Study 2: Algorithm Validation Study

#### Sample and Sampling

From each of the 2 university hospital departments and for the entire rural hospital, 100 patients were randomly selected, for a total sample of 300 patients. In order to have enough patients and the same number from every site, patients were selected based on participation in LPZ/ANQ data collection in 2015, 2016, or 2017. Of a total of 942 patients invited to participate, 705 accepted (75%) for the 3 years. Further inclusion criteria were (1) age ≥18 years, (2) closed and completed patient record, and (3) length of stay ≥24 hours (to avoid outpatients). No formal sample size estimation was conducted. Based on the paper of Schwendimann and colleagues [3], a sample size of 300 yielding a 95% confidence interval ranging of 4.65%-10.89% would be expected.

#### Variables and Measurement

Besides the demographic (age, gender) and diagnostic variables (length of stay, main diagnosis), we focused on the in-hospital fall rates recorded by the 3 methods (Table 1). We also registered the time each method required for data collection. For the fall occurrence variable, we used Hauer et al’s [4] definition and the same inclusion and exclusion criteria as those of our algorithm development study.

#### Data Collection and Management

We used data of patients who participated in the LPZ/ANQ survey, which looks retrospectively at the 30 days before the LPZ/ANQ measurement day. (“Did you fall in the previous 30 days?”) A manual chart review of electronic health records of the 300 patients was carried out using the various electronic health record systems. The electronic health records included patient demographics, diagnoses, clinical data, laboratory results, order entry, reports, and narrative notes.

The manual chart review of the 300 electronic health records was performed by a researcher (ED, one of the first authors) with 5 years of clinical experience in internal medicine as well as knowledge of the hospital’s records. Physicians’ and nurses’ progress notes, physicians’ discharge summaries and nurses’ anamneses of every chart were reviewed. To explore the reliability of ED’s manual chart review, the electronic health records of 20 patients for each of the 3 samples from the 2 departments and the rural hospitals site (20% of the overall sample) were double-reviewed by a clinical nurse specialist with at least 5 years of experience in each respective setting. We obtained a Cohen κ of 0.87, indicating good interrater reliability. Finally, the electronic health record fall detection algorithm was applied by the other first author (BS, informatics nurse) to the full 300-patient sample.

### Algorithm Development and Validation Studies (Study 1 and Study 2)

#### Ethical Considerations

Ethical approval for this study was obtained from the regional Ethics Committee of Bern (development study: 2016-01720; validation study: 2018-01250). Participants of both studies gave informed consent. For data management in both studies, SharePoint (Microsoft Inc) was used. After the merging of the study sample, the patients’ identification numbers were removed and the patients were coded from 1 to 240 and from 1 to 300 for the development and validation studies, respectively. To minimize bias, ED and BS conducted their analyses independently.

#### Data Analysis

R statistical software was used in Windows for all analyses [24]. Version 3.2.4 of the tm package [25] was used for the text-mining part of the algorithm development; version 3.2.5 was used for all other analyses.

For both the development and validation studies, descriptive analyses with means and percentages were conducted for 5 variables: age, gender, length of stay, main diagnosis of the patients, and time for data collection. To gauge diagnostic accuracy, true positive, false positive, false negative, and true negative fall rates were determined. Furthermore, sensitivity, specificity, positive predictive value, and negative predictive value were calculated for each detection method.

Initially the Global Trigger Tool manual method was considered the gold standard for fall detection [10]. However, we recognized that our algorithm detected valid cases that the manual Global Trigger Tool method did not. For example, where patient records are more extensive due to longer hospitalization, reviewer fatigue can lead to inpatient fall events going unnoticed. Therefore, to test the accuracy in the first study, we created a pseudo gold standard by combining the results of the manual Global Trigger Tool study with those of incident reporting and the electronic health record algorithm in the first study and manual chart review, LPZ/ANQ patient reports, and the algorithm in the second study. For both studies, cases with differences between measures (ie, fall in one method versus nonfall in another) were discussed by ED and BS until an agreement was reached.

## Results

### Study 1: Algorithm Development

#### Descriptive Analysis

The mean patient age was 69.3 years (range 18-103). The mean length of stay for patients with fall events of 24.1 (SD 17.6 days) was longer that of the overall study population 13.8 (SD 11.6) days. The study population’s main diagnoses were neurological diseases (48/240, 20.0%), sepsis (37/240, 15.0%), infectious diseases (32/240, 13.3%), and neoplasms (28/240, 11.7%).

#### Diagnostic Accuracy

We report the overall results of the development and validation data sets together (n=240). Twenty fall events were identified by our first composite gold standard and 19 by the development algorithm (sensitivity 95%). The manual Global Trigger Tool method resulted in 18 true positives (90%), whereas incident reporting produced 14 (67%). The manual Global Trigger Tool method and incident reporting produced no false positives, whereas the algorithm resulted in 19 (negative predictive value 99%; positive predictive value 50%); however, most of these related to preadmission fall events: only 2 had no relation to fall events. As noted above, though, while 20 inpatient falls were detected by at least one of the commonly employed methods, the algorithm identified one more legitimate event than the manual Global Trigger Tool method; incident reporting missed 6. For more detailed information see Table 2.

**Table 2:** Diagnostic accuracy results of the comparison between algorithm and all other detection methods in the development and validation studies.

Method			True positive, n		False positive, n		True negative, n		False negative, n		Sensitivity, %		Specificity, %		Positive predictive value, %		Negative predictive value, %
**Development study, development data set (n=120)**																	
	Algorithm	11		10		99		0		100		91		52		100	
	Manual GTT	9		0		109		2		82		100		100		98	
	Incident reporting	7		0		109		4		64		100		100		96	
**Development study, testing data set (n=120)**																	
	Algorithm	8		9		101		2		80		92		47		98	
	Manual GTT	9		0		110		0		100		100		100		100	
	Incident reporting	7		0		110		3		70		100		100		97	
**Validation study (n=298)**																	
	Algorithm	15		17		266		0		100		94		47		100	
	Manual chart review	14		0		283		1		93		100		100		99	
	ANQ^b^ measure	5		0		283		10		33		100		100		97	

^a^GTT: Global Trigger Tool.

^b^ANQ: Swiss National Association For Quality Development in Hospitals and Clinics.

### Study 2: Algorithm Validation

#### Descriptive Analysis

Two patients were excluded because they were minors (<18 years), reducing the total sample to 298 adult inpatients (age: mean 65.3, SD 18.0 years; length of stay: mean 12.1, SD 13.2 days), of which 152 (51.0%) were female (153/298). The most common diagnoses were cardiac (170/298, 57.0%), musculoskeletal (165/298, 55.4%), and endocrine diseases (88/298, 29.5%). The demographics of patients with in-hospital falls versus those without falls did not show any significant differences; however, patients with falls stayed longer in hospital (mean 22.6, SD 19.0 days versus mean 11.5, SD 12.6; *P*=.03). For the manual chart review, ED spent roughly 54 hours (time per record: mean 10.8 minutes).

#### Diagnostic Accuracy

The pseudo gold standard detected 15 falls over the 3606 patient-days (4.16 falls per 1000 patient days) covered by the data period for our study sample (2015-2017). The algorithm recognized all 15 fall events (sensitivity 100%), and the manual chart review identified 14 fall events (93%), whereas the ANQ measure identified only 5 (33%). The algorithm produced no false negatives but 17 false positives, leading to a negative predictive value of 100% and a positive predictive value of 47%. For more detailed information see Table 2.

## Discussion

### Principal Findings

For this study, we first developed an electronic health record algorithm in a single-site sample of 240 patients (development study). We then validated the electronic health record algorithm in a 298-patient sample in 3 departments on 2 sites (validation study). From an epidemiological point of view, the fall rates of 8.3 (development study) and 4.2 (validation study) per 1000 patient-days fit within the range of 2.2-8.9 per 1000 patient-days for Switzerland reported elsewhere [2,3]. For both of our samples, the electronic health record algorithm showed very high sensitivity (95% and 100%), as confirmed by a pseudo gold standard combining the Global Trigger Tool, chart review, voluntary incident reporting, and patient reports of falls. Incident reporting achieved a sensitivity of 67%, the Global Trigger Tool achieved a sensitivity of 90%, manual chart review achieved a sensitivity of 93%, and the patient-reported method of the LPZ/ANQ achieved a sensitivity of only 33%. In the validation study, we found the algorithm’s specificity decreased to 94%, reflecting 17 false positives.

Although the Global Trigger Tool in the development study and manual chart review for falls in the validation study are viewed as the most sensitive methods to identify adverse events [6], our electronic health record algorithm performed slightly better than both, identifying one additional fall in our sample. Unlike manual chart review, the electronic health record algorithm automatically retrieves and evaluates fall cases and is not prone to the subjective weaknesses of manual review, including shortfalls of time, training, or stamina [17-21].

The algorithm’s main disadvantage is its tendency to flag false positive cases, which reduced its positive predictive value to 50% in the development study and 47% in the validation study. All other methods shared a positive predictive value of 100%, as they produced no false positives.

Looking more closely at our algorithm’s false positives, we found that all indicated actual falls, but that the falls had occurred before admission; in several cases, falls were even the reason for admission. While the presence of false positives necessitates further manual screening, the time and effort that this requires is far less than that required for full manual chart review or application of the Global Trigger Tool. For example, while our validation study required 3218.4 minutes (298 patient records × 10.8 minutes) for full manual chart review, based on the mean time spent to review each case, identifying the 17 false positives took only 345.6 minutes ((17 patient records + 15 patient records) × 10.8 minutes)—roughly an 89% reduction.

Additionally, while we will continue to adjust the algorithm to distinguish between preadmission and inpatient falls, a history of falls is an extremely important fall risk indicator [26,27]: identifying any falls will contribute to fall prevention [3,28]. Nevertheless, the argument in favor of using our algorithm for inpatient fall detection is a matter of efficiency: instead of requiring 10 minutes per case for a full manual review or 20 to apply the Global Trigger Tool, the algorithm requires only a few seconds, after which only the positive results (roughly 11% of the full case number) require review. The algorithm can detect falls near real time and can be used on a daily or weekly basis while the patient is still in hospital. Detection during the patient stay is probably less relevant for the clinical management of individual patients but could provide a management tool to identify areas with unusually high fall incidence, which could be supported by additional resources.

Another vitally important value this study provides is the transferability of the electronic health record algorithm to other departments and institutions with a broad range of electronic health record systems. As data sources, electronic health records are rich but often somewhat chaotic, adding to the complexity of adverse event detection. Terms used to report fall events vary between settings, which could limit an algorithm’s performance [29]. However, in our validation study, the same unmodified version of our algorithm returned excellent results in 3 clinical departments on 2 sites (using 2 electronic health record systems) [30-32].

In contrast, the LPZ/ANQ measure identified only 5 of 15 confirmed fall events. Underreporting and nonreporting are possible and frequent with this method, as it depends on each patient’s capacity to remember and report fall events. It is well-established that retrospective reports depend on the cognitive, mental, and physical condition of the patient at the moment of the interview [33]. In addition, a patient might not know what qualifies as a fall (such as when the patient begins to fall and is assisted to the ground by another person). The low count of the LPZ/ANQ-measure is also explained by their 1-point prevalence measurement, which only captures falls from admission until the LPZ/ANQ measurement date. Because the prevalence measure can occur on any day of the hospital stay of the patients only half of the length of stay will be taken into account. If falls occur evenly distributed throughout the hospital stay the number of falls detected is also cut in half. As the LPZ/ANQ detected only about one-third or less of our sample’s in-hospital falls (5 versus 15), we can only conclude that it cannot provide robust prevalence rates. Although it is not unreasonable to assume that the described biases will be similar across hospitals [34], the extent of this method’s underreporting and the high cost of each primary data collection on a national scale raise doubts about its overall value.

The use of highly sensitive electronic health record algorithms to detect adverse events and small-scale validation studies such as ours opens up at least 2 productive pathways for future research. First, the current algorithm allows expanding data sets by manual screening of the cases identified by the algorithm. With the same resources (validation study 300 records), we are now able to screen 3000 records. Such a data set could then be used for refining the algorithm to improve the specificity but also allows for conducting substantive analysis (eg, on risk factors of falls). Second, the study design could serve as a template for developing additional electronic health record adverse event detection algorithms. This is particularly interesting when exploring the association of structural and process measures with quality of care outcomes in a causal inference data-fusion framework [35]. Data fusion in this context would allow using data from a validation study to overcome measurement error in routine electronic health record data.

### Limitations

This study is subject to several notable limitations. First, the quality of any algorithm’s results cannot surpass that of the documentation upon which it is based, that is, the quality and the completeness of the documentation define the limits of the algorithm’s performance [17,29]. Therefore, heavy workloads, which influence documentation quality, also influence our algorithm’s capacity to detect falls. In case of acute situations, documentation is often done on paper and later transcribed to the electronic health record, which can lead to missing information [17]. While these limitations also apply to other fall detection methods [29], both the Global Trigger Tool and manual review draw their data from broader sources, which may increase the chances of detecting traces of an event. The small sample size for both the development and the validation studies, as well as the lack of a true gold standard represent other limitations.

Finally, we based our selection of patients on the LPZ/ANQ measure, which suffers from selection bias: patients who did not speak one of the Swiss national languages, had cognitive limitations (eg, dementia or delirium), were dying or in unstable states were excluded. For example, in our validation study, only 75% of the patients participated in the LPZ/ANQ data collection.

### Conclusions

For this study, we successfully developed and evaluated a newly developed algorithm for fall detection, which we tested in the electronic health records of 3 different departments situated on 2 sites. Weighing the advantages and disadvantages of the different methods used in this study, our algorithm is extremely attractive: of all the methods employed in the tests, our fall detection algorithm offered the highest sensitivity with by far the smallest time investment. And although it produced false positives, thereby necessitating a manual chart review of all identified cases, the overall time investment and sensitivity were roughly 90% better than those for the other methods with comparable sensitivity. Applied in near real time, the algorithm can record in-hospital fall events at least as effectively as manual chart review or the Global Trigger Tool but requires a small fraction of the time or human resources demanded by either. Not only will this algorithm contribute to a better understanding of inpatient falls, it will also highlight fall-influencing factors, thereby helping identify the patients with the highest risk of falls, all of which will promote development and targeting of preventive interventions. Each implementation of this algorithm will offer an opportunity to fine-tune it, particularly to distinguish between inpatient and preadmission falls (false positives). Further research on this algorithm using a larger data sample or using the algorithm on a weekly basis can generate further data and feedback in order to improve it.

## Appendix

Multimedia Appendix 1 Variable details.

Multimedia Appendix 2 Patient record selection criteria.

## Abbreviations

ANQ: Swiss National Association For Quality Development in Hospitals and Clinics
LPZ: Landelijke Prevalentiemeting Zorgproblemen

## References

[1] Bouldin ELD, Andresen EM, Dunton NE, Simon M, Waters TM, Liu M, Daniels MJ, Mion LC, Shorr RI (2013). Falls among adult patients hospitalized in the United States: prevalence and trends. J Patient Saf.

[2] Halfon P, Eggli Y, Van Melle G, Vagnair A (2001). Risk of falls for hospitalized patients: a predictive model based on routinely available data. J Clin Epidemiol.

[3] Schwendimann R, Bühler H, De Geest S, Milisen K (2006). Falls and consequent injuries in hospitalized patients: effects of an interdisciplinary falls prevention program. BMC Health Serv Res.

[4] Hauer K, Lamb SE, Jorstad EC, Todd C, Becker C, PROFANE-Group (2006). Systematic review of definitions and methods of measuring falls in randomised controlled fall prevention trials. Age Ageing.

[5] Cina-Tschumi B, Schubert M, Kressig RW, De Geest S, Schwendimann R (2009). Frequencies of falls in Swiss hospitals: concordance between nurses' estimates and fall incident reports. Int J Nurs Stud.

[6] Murff HJ, Patel VL, Hripcsak G, Bates DW (2003). Detecting adverse events for patient safety research: a review of current methodologies. J Biomed Inform.

[7] Blegen MA, Vaughn T, Pepper G, Vojir C, Stratton K, Boyd M, Armstrong G (2004). Patient and staff safety: voluntary reporting. Am J Med Qual.

[8] Evans SM, Berry JG, Smith BJ, Esterman A, Selim P, O'Shaughnessy J, DeWit M (2006). Attitudes and barriers to incident reporting: a collaborative hospital study. Qual Saf Health Care.

[9] Griffin F, Resar RK (2009). IHI Global Trigger Tool for measuring adverse events. IHI Innovation Series White Paper.

[10] Classen DC, Resar R, Griffin F, Federico F, Frankel T, Kimmel N, Whittington JC, Frankel A, Seger A, James BC (2011). 'Global trigger tool' shows that adverse events in hospitals may be ten times greater than previously measured. Health Aff (Millwood).

[11] Doupi P, Svaar H, Bjørn B, Deilkås E, Nylén U, Rutberg H (2015). Use of the Global Trigger Tool in patient safety improvement efforts: Nordic experiences. Cogn Tech Work.

[12] Govindan M, Van CAD, Nelson EC, Kelly-Cummings J, Suresh G (2010). Automated detection of harm in healthcare with information technology: a systematic review. Qual Saf Health Care.

[13] Landelijke Prevalentiemeting Zorgproblemen (LPZ).

[14] Swiss National Association For Quality Development in Hospitals and Clinics (ANQ).

[15] Murff HJ, Patel VL, Hripcsak G, Bates DW (2003). Detecting adverse events for patient safety research: a review of current methodologies. J Biomed Inform.

[16] Musy SN, Ausserhofer D, Schwendimann R, Rothen HU, Jeitziner M, Rutjes AW, Simon M (2018). Trigger Tool–Based Automated Adverse Event Detection in Electronic Health Records: Systematic Review. J Med Internet Res.

[17] Li Q, Melton K, Lingren T, Kirkendall ES, Hall E, Zhai H, Ni Y, Kaiser M, Stoutenborough L, Solti I (2014). Phenotyping for patient safety: algorithm development for electronic health record based automated adverse event and medical error detection in neonatal intensive care. J Am Med Inform Assoc.

[18] Melton GB, Hripcsak G (2005). Automated detection of adverse events using natural language processing of discharge summaries. J Am Med Inform Assoc.

[19] Penz JFE, Wilcox AB, Hurdle JF (2007). Automated identification of adverse events related to central venous catheters. J Biomed Inform.

[20] Murff HJ, FitzHenry F, Matheny ME, Gentry N, Kotter KL, Crimin K, Dittus RS, Rosen AK, Elkin PL, Brown SH, Speroff T (2011). Automated identification of postoperative complications within an electronic medical record using natural language processing. JAMA.

[21] Toyabe S (2012). Detecting inpatient falls by using natural language processing of electronic medical records. BMC Health Serv Res.

[22] Grossmann N, Gratwohl F, Musy SN, Nielen NM, Simon M, Donz J (2019). Describing adverse events in medical inpatients using the Global Trigger Tool. Swiss Med Wkly.

[23] Soop M, Fryksmark U, Köster M, Haglund B (2009). The incidence of adverse events in Swedish hospitals: a retrospective medical record review study. Int J Qual Health Care.

[24] The R Project for Statistical Computing. The R Foundation, Vienna, Austria; 2020.

[25] Feinerer I, Hornik K, Meyer D (2008). Text Mining Infrastructure in. J. Stat. Soft.

[26] Müller R, Halfens R, Schwendimann R, Müller M, Imoberdorf R, Ballmer PE (2009). Risikofaktoren für Stürze und sturzbedingte Verletzungen im Akutspital – Eine retrospektive Fall-Kontroll-Studie. Pflege.

[27] Tzeng H, Yin C (2013). Frequently observed risk factors for fall-related injuries and effective preventive interventions: a multihospital survey of nurses' perceptions. J Nurs Care Qual.

[28] Schwendimann R (2000). [Prevention of falls in acute hospital care. Review of the literature]. Pflege.

[29] Halfon P, Staines A, Burnand B (2017). Adverse events related to hospital care: a retrospective medical records review in a Swiss hospital. Int J Qual Health Care.

[30] Hripcsak G, Bakken S, Stetson PD, Patel VL (2003). Mining complex clinical data for patient safety research: a framework for event discovery. J Biomed Inform.

[31] Murphy DR, Thomas EJ, Meyer AND, Singh H (2015). Development and Validation of Electronic Health Record-based Triggers to Detect Delays in Follow-up of Abnormal Lung Imaging Findings. Radiology.

[32] Liao KP, Ananthakrishnan AN, Kumar V, Xia Z, Cagan A, Gainer VS, Goryachev S, Chen P, Savova GK, Agniel D, Churchill S, Lee J, Murphy SN, Plenge RM, Szolovits P, Kohane I, Shaw SY, Karlson EW, Cai T (2015). Methods to Develop an Electronic Medical Record Phenotype Algorithm to Compare the Risk of Coronary Artery Disease across 3 Chronic Disease Cohorts. PLoS One.

[33] ANQ (2014). Überprüfung ANQ-Messplan auf Vollständigkeit und Relevanz: Kurzfassung zum 2. Teil des Forschungsberichts des ISGF inkl. Identifikation von Handlungsoptionen.

[34] ANQ (2018). Auswertungskonzept ANQ: Nationale Prävalenzmessung Sturz & Dekubitus Erwachsene und Dekubitus Kinder. www.anq.ch.

[35] Bareinboim E, Pearl J (2016). Causal inference and the data-fusion problem. Proc Natl Acad Sci USA.

