# Supplementary material for: Automated Fall Detection Algorithm With Global Trigger Tool, Incident Reports, Manual Chart Review, and Patient-Reported Falls: Algorithm Development and Validation With a Retrospective Diagnostic Accuracy Study
Source: J Med Internet Res. 2020 Sep 21;22(9):e19516. doi: 10.2196/19516 (PMC7536608; doi:10.2196/19516)
Supplement: Multimedia Appendix 1 [file jmir_v22i9e19516_app1.docx]

**Appendix 1**

Table 4. Details of the variables of the study.

| Variable | Method and data sources | Method and data sources | Method and data sources | Information | Original variables names in ANQ |
| --- | --- | --- | --- | --- | --- |
|  | Algorithm | Manual chart review | LPZ/ANQ |  |  |
|  |  |  |  |  |  |
| Age |  |  | x |  | 2015: Codebook LPZ2015NOV: variable *Age* (position 137) |
|  |  |  |  |  | 2016: Codebook CH2016NOV: variables *Date of admission* (71) – *Date of birth* (69) = age |
|  |  |  |  |  | 2017: Codebook CH2017NOV: variables *Date of admission* (22) – *Date of birth* (26) = age |
| Gender |  |  | x |  | 2015: Codebook LPZ2015NOV: variable *Gender/Sex* (138); 1 = male, 2 = female |
|  |  |  |  |  | 2016: Codebook CH2016NOV: variable *Sex* (70); 1 = male, 2 = female |
|  |  |  |  |  | 2017: Codebook CH2017NOV: variable *Sex* (25); 1 = male, 2 = female |
| Length of stay |  | x |  | Calculated: date of discharge - date of admission | - |
| Main diseases |  |  | x |  | 2015: Codebook LPZ2015NOV: variables *diagn_[…]*, 146-168; 0 = no, 1 = yes |
|  |  |  |  |  | 2016: Codebook CH2016NOV: variables *G_diag_[…]*, 73-91; 0 = no, 1 = yes |
|  |  |  |  |  | 2017: Codebook CH2017NOV: variables *G_diag_[…]*, 28-46; 0 = no, 1 = yes |
| Presence of fall | x | x | x |  | 0 = no fall, 1 = fall |
| Fall rates | x | x | x | Calculated | - |
| Time for data collection | x | x | x | Calculated | - |

Note. a) A = Algorithm; b) M = manual chart review; c) LPZ = LPZ/ANQ-measure
