# Supplementary material for: Automated Fall Detection Algorithm With Global Trigger Tool, Incident Reports, Manual Chart Review, and Patient-Reported Falls: Algorithm Development and Validation With a Retrospective Diagnostic Accuracy Study
Source: J Med Internet Res. 2020 Sep 21;22(9):e19516. doi: 10.2196/19516 (PMC7536608; doi:10.2196/19516)
Supplement: Multimedia Appendix 2 [file jmir_v22i9e19516_app2.docx]

**Appendix 2**

Table 5. List of criteria terms for inclusion and for exclusion of patient records in the algorithm.

| Exclusion-Criteria | Exclusion-Criteria | Inclusion-Criteria |
| --- | --- | --- |
| *sturzangst* | *sturz- und weglaufgefährdet* | *am boden* |
| *angst* stürzt* | *sturz zu hause* | *ausgerutscht* |
| *angst * sturz* | *stürzen abgehalten* | *sturz* |
| * angst vor * stürzen*' | *sturzgefahr* | *stürz* |
| *sturzprophylaxe* | *sturzgefährde* | *synkop* |
| *abgestürzt* | *sturzgefährdung* |  |
| *alkoholabstürze* | *stürzt * fast* |  |
| *am boden * *ml* | *stürzte beinahe* |  |
| *angst * synkopieren* | *synkopen * vermeiden* |  |
| *beinahe gestürzt* | *wäre *stürzt * |  |
| *decke * am boden* | *zu hause* |  |
| *eintritt* | *zugewiesen * |  |
| *fast gestürzt* |  |  |
| *fats gestürz* |  |  |
| *fuss *am boden* |  |  |
| *füsse *am boden* |  |  |
| *gefährdung zu stürzen* |  |  |
| *häuslicher sturz * |  |  |
| *hindernisse am boden* |  |  |
| *kein * synkop* |  |  |
| *kein erneutes sturzeregniss * |  |  |
| *kein* synkope* |  |  |
| *kissen * am boden* |  |  |
| *klingel rausgerutscht* |  |  |
| *konsilium * |  |  |
| *ml am boden* |  |  |
| *nicht gestürzt* |  |  |
| *notfall* |  |  |
| *poliklinik* |  |  |
| *präsynkope* |  |  |
| *präsynkope* |  |  |
| *pvk * herausgerutscht* |  |  |
| *sprechstunde* |  |  |
| *st. n. sturz* |  |  |
| *stationäre aufnahme* |  |  |
| *status nach sturz* |  |  |
| *sturz * vermeiden* |  |  |
